# Remodeling of the Microvasculature: May the Blood Flow Be With You

**DOI:** 10.3389/fphys.2020.586852

**Published:** 2020-10-15

**Authors:** Ricardo Santamaría, María González-Álvarez, Raquel Delgado, Sergio Esteban, Alicia G. Arroyo

**Affiliations:** ^1^Department of Vascular Pathophysiology, Centro Nacional de Investigaciones Cardiovasculares (CNIC), Madrid, Spain; ^2^Department of Molecular Biomedicine, Centro de Investigaciones Biológicas Margarita Salas (CIB-CSIC), Madrid, Spain

**Keywords:** microvascular remodeling, capillary pruning, capillary splitting, blood flow, shear stress, endothelial cells, 3D-confocal microscopy, microfluidics

## Abstract

The vasculature ensures optimal delivery of nutrients and oxygen throughout the body, and to achieve this function it must continually adapt to varying tissue demands. Newly formed vascular plexuses during development are immature and require dynamic remodeling to generate well-patterned functional networks. This is achieved by remodeling of the capillaries preserving those which are functional and eliminating other ones. A balanced and dynamically regulated capillary remodeling will therefore ensure optimal distribution of blood and nutrients to the tissues. This is particularly important in pathological contexts in which deficient or excessive vascular remodeling may worsen tissue perfusion and hamper tissue repair. Blood flow is a major determinant of microvascular reshaping since capillaries are pruned when relatively less perfused and they split when exposed to high flow in order to shape the microvascular network for optimal tissue perfusion and oxygenation. The molecular machinery underlying blood flow sensing by endothelial cells is being deciphered, but much less is known about how this translates into endothelial cell responses as alignment, polarization and directed migration to drive capillary remodeling, particularly *in vivo*. Part of this knowledge is theoretical from computational models since blood flow hemodynamics are not easily recapitulated by *in vitro* or *ex vivo* approaches. Moreover, these events are difficult to visualize *in vivo* due to their infrequency and briefness. Studies had been limited to postnatal mouse retina and vascular beds in zebrafish but new tools as advanced microscopy and image analysis are strengthening our understanding of capillary remodeling. In this review we introduce the concept of remodeling of the microvasculature and its relevance in physiology and pathology. We summarize the current knowledge on the mechanisms contributing to capillary regression and to capillary splitting highlighting the key role of blood flow to orchestrate these processes. Finally, we comment the potential and possibilities that microfluidics offers to this field. Since capillary remodeling mechanisms are often reactivated in prevalent pathologies as cancer and cardiovascular disease, all this knowledge could be eventually used to improve the functionality of capillary networks in diseased tissues and promote their repair.

## Dynamic Microvascular Remodeling in Physiology and Pathophysiology

**FIGURE 1:**
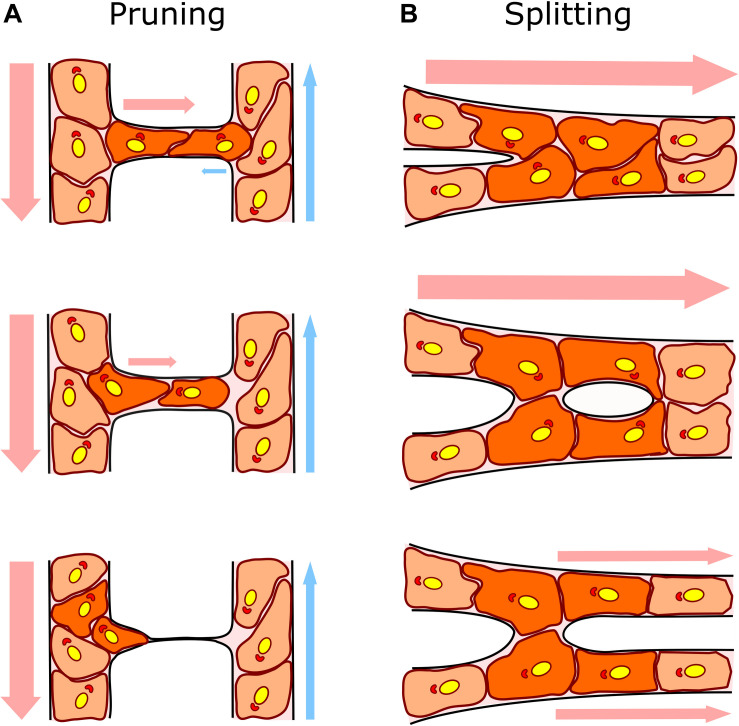
Capillary remodeling by pruning/regression and splitting/duplication. **(A)** In capillary pruning the poorly perfused vessel is selected to regress, its lumen collapses and the endothelial cells inside (dark orange) polarized against flow and migrate toward the higher flow adjacent vessel. **(B)** In capillary splitting, highly perfused vessels vasodilate and endothelial cells nearby the intersections (Y bifurcation; dark orange) reorganize their cytoskeleton, migrate toward the lumen and form an intraluminal pillar that will eventually split the vessel forming two daughter vessels. Arrows indicate the direction and intensity of the blood flow (arterial flow in red and venous flow in blue). Endothelial cell nuclei and Golgi are colored in yellow and red, respectively, to show endothelial cell polarization preferentially against the flow.

The microvasculature constantly adjusts to tissue metabolic demands through functional and structural adaptations (Pries and Secomb, 2014). This is attained by dynamic remodeling of the capillaries preserving and expanding those which are functional and eliminating redundant or poorly efficient ones. We will refer in this review as microvascular or capillary remodeling to the dynamic gain or loss of capillaries for increasing or decreasing the complexity of the microvascular network in order to optimize oxygen and nutrient distribution into the tissue. Two main processes determine microvascular reshaping in several organs and tissues during their growth and development: i) capillary pruning or regression, the elimination of non-functional capillaries to form a functional hierarchically branched network; and ii) duplication of highly perfused capillaries which results in the quick expansion of the microvasculature. Duplication can occur via intussusceptive angiogenesis, recognized as the division of a large lumen within a sinus, or by splitting in tubular capillaries, the process in which we will mainly focus this review (Figure 1).

Once immature vascular plexuses are formed mostly by sprouting angiogenesis, capillary remodeling becomes essential to preserve tissue homeostasis in physiological contexts during development but also in adult tissues. Capillary regression takes place in all developmental stages during embryogenesis after the formation of the first primitive vasculature as in the chicken yolk sac and limb embryo (Feinberg et al., 1986; Lee et al., 2011) or the brain and intersegmental vessels in the zebrafish (Chen et al., 2012; Kochhan et al., 2013; Franco et al., 2015), but also right after birth like in the postnatal mouse retina (Connolly et al., 1988). Vascular regression can also be associated not only to optimization of the blood flow network but also to the elimination of unneeded tissue like in hyaloid regression after birth (Lang et al., 1994; Ito and Yoshioka, 1999; Lobov et al., 2005) or in involution of post-lactation mammary gland and ovarian luteolysis in the adult (Goede et al., 1998; Andres and Djonov, 2010); in these contexts endothelial cell apoptosis is considered the main mechanism and will not be discussed further in this review (Watson et al., 2017). Capillary duplication promotes expansion of the microvasculature during embryo and organ development as observed in the chicken chorioallantoic membrane (Patan et al., 1993) and in a variety of tissues after birth as the lung, heart, intestine, liver or kidney (Patan et al., 1992). It also contributes to capillary growth upon increasing tissue demands as in the exercising skeletal muscle (Prior et al., 2003; Mentzer and Konerding, 2014). Notably these vascular reshaping mechanisms are often reactivated or defective in prevalent pathologies leading generally to a dysfunctional microvasculature which aggravates damage and hinders tissue repair. Although altered capillary pruning has been described in tumors (Holash et al., 1999), it may also occur in ischemic disorders as myocardial infarction (Luttun et al., 2002; Gkontra et al., 2019), hypertension (Boudier, 1999), and age-related neurodegeneration or Alzheimer’s disease (Wu et al., 2005; Bell and Zlokovic, 2009; Sagare et al., 2012) exacerbating the hypo-perfusion of the damaged tissue. Capillary splitting has been observed among others in inflammatory bowel disease (Mori et al., 2005; Ravnic et al., 2007; Esteban et al., 2020), tumors (Patan et al., 1996b; Ribatti and Djonov, 2012), lung dysplasia of prematurity (De Paepe et al., 2015, 2017) and other syndromes (Giacomini et al., 2015) contributing to disease progression. Impaired endothelial cell responses involved in microvascular remodeling can also result in the persistence or arteriovenous shunts, the basis for arteriovenous malformations (AVM) (Red-Horse and Siekmann, 2019).

Capillary remodeling events are dynamic, occurring in hours to a few days and involve active endothelial cell rearrangements and quick morphological changes of the microvasculature, often without compromising its integrity since the basement membrane is preserved (Ricard and Simons, 2015). The current model for capillary pruning proposes a multi-staged model to explain the dynamics of the vessel regression process observed in the postnatal mouse retina and intersegmental vessels in zebrafish (Franco et al., 2015, 2016; Franco and Gerhardt, 2017). Firstly, there is a blood flow-driven selection of the regressing vascular branch which triggers the subsequent morphological alterations. The selected vessel receiving low flow constricts leading to lumen stenosis or even collapse. Then, endothelial cells retract along with junctional remodeling and migrate intraluminally from the regressing segment to the higher-flow adjacent vessel (Franco et al., 2015; Barbacena et al., 2016). The final resolution step involves the complete integration of endothelial cells from the regressing branch into the neighbor vessel leaving an empty basement membrane surrounded by pericytes which is called “empty sleeve” and considered a hallmark of vessel regression (Barbacena et al., 2016). The number of regressed segments increased only slightly and did not accumulate from P4 to P8 during remodeling of the postnatal retinal microvasculature indicating that in spite of the limitation of the analysis in still images, these pruning events seem dynamic and with a limited lifetime (Franco et al., 2015). Accordingly, live microscopy of the intersegmental vessels of zebrafish embryos showed that endothelial cell dynamics during segment regression occur in about 24 h (Franco et al., 2015).

In the context of capillary duplication by splitting or intussusceptive angiogenesis observed for example in the chicken chorioallantoic membrane, the rat skeletal muscle and the mouse inflamed intestine, the division of the vascular plexus takes place through the formation and expansion of intraluminal pillars. Increased blood flow in the capillary bed causes vasodilation of the blood vessels. The endothelial cells of the vessel reorganize their cytoskeleton, develop luminal filopodia mostly from the inter-junctional area and migrate toward the vascular lumen until they join the opposite side and rearrange their junctions (Patan et al., 1996a; Burri et al., 2004; Williams et al., 2006a). The cells form a cell bridge in the center of the vessel, rich in parallel actin filaments. These steps in the nascent pillar have been visualized by microscopy techniques, but it remains unknown how filopodia grow and fuse under high blood flow that would impose a mechanical barrier to the process. Pillars form in vessels with an intact basement membrane (Mentzer and Konerding, 2014), and subsequently, a 1–5 μm-pore forms in the center of the capillary which is invaded by surrounding tissue pericytes and myofibroblasts. These cells deposit collagen fibers stabilizing the formation of the pillar. Finally, the pillars grow in diameter and join other pillars splitting the capillary into two parallel vessels in the form of loops and duplications (Burri et al., 2004; Karthik et al., 2018). Of note, pillar formation in the chorioallantoic membrane initiated 40 min to few hours after vasodilation and in the inflamed ear model full capillary splitting was achieved in 3 days (Styp-Rekowska et al., 2011). Recently a pioneer study has analyzed the dynamics of vessel splitting by live microscopy imaging in the caudal vein plexus of zebrafish and showed that pillar formation and fusion can occur in just a few hours (Karthik et al., 2018). In this study, new data obtained by 3D reconstruction of scanning electron microscopy images have shown with unprecedented resolution the process of formation of intraluminal pillars and their fusion and highlighted the need of understanding the dynamics and cellular events of the loss of endothelial cell polarity, cytoskeleton rearrangement and establishment of new endothelial cell junctions at the pillar (Karthik et al., 2018).

## Blood Flow-Driven Capillary Regression and Splitting: Two Sides of the Same Coin?

**FIGURE 2:**
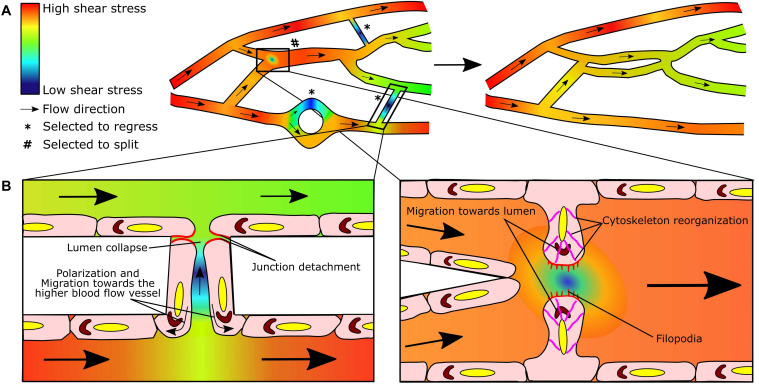
Blood flow-driven capillary pruning and splitting: two sides of the same coin. **(A)** Heterogeneous flow distribution in dynamic microvascular networks results in shear stress gradients (left). As a consequence, lower perfused segments will be selected to regress (*) and highly perfused segments to split (#) in order to redistribute flow in a more efficient manner in the remodeled network (right). **(B)** Endothelial cells sense the blood flow and shear stress gradients and respond by elongating and aligning in the direction of flow. In the case of pruning, endothelial cells in the regressing segment (left panel) detach from their neighbors (red) during lumen collapse, polarize (Golgi in brown) and migrate toward the region of higher flow in the adjacent vessel (curved arrows). In capillary splitting (right panel), endothelial cells close to shear stress gradient at the bifurcation, reorganize their cytoskeleton (pink), protrude luminal filopodia (red), and polarize (Golgi in brown) and migrate toward the lumen to form the intraluminal pillar; note the area drawn with low blood flow/shear stress at the nascent pillar which could be permissive for filopodia growth and fusion. Heat map colors indicate the predicted/simulated shear stress values in the modeled microvascular network. Straight arrows indicate the flow direction. Adapted from Chen et al. (2012).

The dynamic nature of microvascular remodeling closely reflects the dynamically changing nature of blood flow, constantly responding to physicochemical cues in the tissues (Pries and Secomb, 2014). Pioneer studies in the embryo yolk sac established the essential role of blood flow in vascular remodeling (le Noble et al., 2004) and put the foundations about the dynamic responses induced by blood flow in endothelial cells as their directed migration from small capillaries to larger arteries (Udan et al., 2013). In addition to influencing angiogenic endothelial cell sprouting (Campinho et al., 2020), blood flow governs microvascular remodeling by promoting pruning of capillaries when not constantly perfused and expanding them when exposed to high flow in order to shape the microvascular network for optimal blood flow distribution (Figure 2A). However, the concept that low flow drives pruning and high flow triggers splitting is an oversimplification since it is not the magnitude but the gradients of blood flow that rule both remodeling processes as discussed in more detail in the following sections.

### Blood Flow Basics

Blood flow refers to the movement of the blood through the vessels. Flow is typically characterized based on certain physical parameters yielding different types of flow (Chiu and Chien, 2011). The flow is considered laminar, that is smooth and continuous, when all the fluid particles flow in parallel fluid layers. Otherwise, flow is defined as turbulent when it experiences irregularities and chaotic motion as it occurs at high velocities or in certain geometries such as big curvatures, bifurcations, anastomoses or stenotic sites. In general, the microcirculation responds to a steady and laminar flow. However, this assumption cannot be made in larger vessels like arteries where the flow is pulsatile due to the heart beat. Another type of flow observed in the vasculature and maybe relevant for remodeling events is reciprocating flow. In reciprocating or oscillatory flow, the velocity oscillates back and forth with a given frequency, resulting in a minimal forward net blood flow. Impinging flow can be found mainly at arterial bifurcation apices. This type of flow has classically a T-shaped geometry, where the inlet with high velocity collapses against a wall generating a stagnation point (Ostrowski et al., 2014).

Wall shear stress (referred as shear stress hereon) is the force per unit area acting on the vessel wall performed by the blood flowing through the vessel. It has pressure units (Pascal, Pa, in IS; in vascular research dyn/cm^2^). This force is tangential and depends on two parameters: the viscosity of blood and the rate at which the velocity of blood changes along the vessel radius (shear rate). The shear rate could be in theory correlated with vessel diameter, but only taking into consideration the instantaneous volumetric flow rate and velocity field through that vessel. In addition, since the blood is in reality a non-Newtonian fluid, its viscosity will change depending on this shear rate (Eckmann et al., 2000). Recent computational efforts have also shown the essential role of circulating cells, often disregarded in theoretical *in silico* simulations, in modulating blood viscosity and regional shear stress (Zhou et al., 2020). Moreover, the geometry of the microvascular network also influences shear stress patterns and values, particularly at curvatures and bifurcations (Chiu and Chien, 2011). Therefore, even though in principle shear stress could be expected to be high in small vessels, in regressing capillaries (with net blood flow practically null) and duplicating capillaries (with altered blood flow related to the geometry), shear stress values are rather low. In fact, shear stress and fluid velocity are simulated together and correlate in the retina and other vascular plexus (Filipovic et al., 2009; Szczerba et al., 2009; Bernabeu et al., 2014). In Table 1 shear stress values at different vascular territories and developmental stages are included as a reference.

**TABLE 1:** Shear stress in microvascular remodeling. Summary of shear stress (SS) values in selected vascular territories under homeostatic and microvascular remodeling conditions *in vivo*. Note that capillary shear stress values obtained by computational approaches are based on theoretical input pressure values into the simulated network and may not accurately reflect *in vivo* physiological values.

Vascular bed	SS (dyn/cm^2^)	Comments	References
**Physiological shear stress values**			
**Human**			
Arteries (various tissues)	1–7		Chiu and Chien (2011)
Veins (various tissues)	0.6–1.1		
Vascular geometries	<4	Arterial branch points and curvatures	
**Mouse**			
Embryo	≈5		Udan et al. (2013)
Retinal Arteriole (p5 and p6)	0–200	Based on computational simulations	Bernabeu et al. (2014), Franco et al. (2015)
Retinal Arteriole (8-12 weeks)	70	Using a network modeling	Ganesan et al. (2010)
Retinal Venule (p5)	0–100	Based on computational simulations	Bernabeu et al. (2014)
Retinal Venule (p6)	0–150	Based on computational simulations	Franco et al. (2015)
Retinal Venule (8–12 weeks)	55	Using a network modeling	Ganesan et al. (2010)
Retinal Capillaries (p5 and p6)	0–200	Based on computational simulations	Bernabeu et al. (2014), Franco et al. (2015)
Retinal vessels order 1–3 (arterial)	40–110	Using a network modeling	Ganesan et al. (2010)
Retinal vessels order 1–3 (venous)	25–90	Using a network modeling	Ganesan et al. (2010)
Postnatal and adult mouse aorta	60–250	Mean SS in postnatal: 140 dyn/cm^2^ Mean SS in adult: 95 dyn/cm^2^	Trachet et al. (2009)
**Zebrafish**			
Caudal Plexus Vein 25–42 hpf	0–22.5	Computational simulations	Karthik et al. (2018)
	0–15	At the pillar/splitting zone	
Intersegmental Vessels 5 dpf	1.2 ± 0.2	Pruning usually occurring	Choi et al. (2017)
**Chicken chorioallantoic membrane (CAM)**			
Chicken CAM Arterioles	4.47 ± 2.7	Mathematical simulations	Maibier et al. (2016)
Chicken CAM Venules	4.65 ± 3.4	Mathematical simulations	
**Rat**			
Skeletal muscle	5.6 ± 0.8		Hudlicka et al. (2006)
**Capillary pruning and splitting shear stress values**			
**Pruning**			
Retina (p6)	0–1	Computational simulation pruned vessel	Bernabeu et al. (2014)
Retina (p6)	≈0	Computational simulation pruned vessel	Franco et al. (2015)
Zebrafish Brain (3 dpf)	0.55 ± 0.05 0.18 ± 0.02	Computational simulation of pruned vessel (2 different events)	Chen et al. (2012)
	1 ± 0.2 1	Computational simulation of unpruned adjacent vessel (2 different events)	
**Splitting**			
Zebrafish CVP (25–42 hpf)	0.4 and 1	Computational simulation after pillar appearance (values of 2 different pillars)	Karthik et al. (2018)
	1.6 and 5	Computational simulation before pillar appearance (linked with above pillars)	
	11	Computational simulation, 5 μm to pillar	
	0.8	Computational simulation in the shortest distance to the same pillar above	
Chick CAM	<0.3 and <0.6	In the zone of the first intravascular pillar and in the interpillar surfaces	Lee et al. (2010)
Murine colitis	15–45	Computational simulations	Filipovic et al. (2009)
Murine colitis (and CAM)	<1	Dead zone where pillars form	
Rat skeletal muscle (2 day-activity)	11.4 ± 1.0	Prior to splitting	Hudlicka et al. (2006)

Shear stress is sensed by the endothelial cells in the inner lining of the vessels and it is a major determinant of endothelial cell behavior (Chiu and Chien, 2011). But it is not only the magnitude of shear stress but its fluctuations that determine the responses of endothelial cells and therefore influence capillary remodeling. Time-related parameters as duration, ramping rate and frequency/oscillations of shear stress have differential impact on endothelial cell morphology and behavior (Yoshino et al., 2017). As an example, long exposure to disturbed instead of laminar flow upregulates pro-inflammatory genes and proliferation, which predisposes to atherosclerosis (Chiu and Chien, 2011; Mack et al., 2017), but short exposure to changing shear stress direction may underlie endothelial cell morphological changes relevant to capillary remodeling (Wang et al., 2013). And abrupt increase in shear stress augments endothelial cell membrane fluidity while gradual increase does not (Butler et al., 2002). Local changes and the 3D geometry of the network also generate shear stress spatial gradients between adjacent or nearby endothelial cells *in vitro* (Mack et al., 2017) or vascular segments *in vivo* (Franco et al., 2015). The pattern of shear stress is different in straight, branched and curved regions of the vasculature (Karino and Goldsmith, 1980; Karino et al., 1987; Nerem, 1993; Colangelo et al., 1994; Chien, 2003) and this can be related with the preference for Y and polygonal geometries in capillary splitting (Filipovic et al., 2009; Karthik et al., 2018) and H or O (loop) geometries in capillary regression (Bernabeu et al., 2014; Barbacena et al., 2016).

### Blood Flow and Capillary Pruning

Early reports pointed to endothelial cell apoptosis as the main actor in vessel regression (Meeson et al., 1999; Hahn et al., 2005; Lobov et al., 2005; Wang et al., 2011; Simonavicius et al., 2012). More recent works in the mouse yolk sac and postnatal retina among others have shown however that blood flow is critical to induce dynamic endothelial cell responses (le Noble et al., 2004; Udan et al., 2013; Franco et al., 2015). In particular, endothelial cells elongate and align along shear stress axis and gradients of shear stress induce endothelial cell polarization and directed migration toward the higher blood flow adjacent vessel during capillary pruning (Franco et al., 2015; Barbacena et al., 2016) (Figure 2B). Moreover, studies such as those performed in the brain vasculature of zebrafish suggest the existence of a flow threshold for vessel regression (Chen et al., 2012).

This new concept establishes that, contrary to expected, capillary endothelial cells are not quiescent, but subjected to flow changes that regulate their polarization and migratory behavior. It has been proposed that differential blood flow/shear stress patterns in juxtaposed vessels drive asymmetries in cellular movements causing endothelial cells to migrate toward high flow neighboring vessels, thus favoring regression and pruning of the low blood flow capillaries (Lucitti et al., 2007; Chen et al., 2012; Kochhan et al., 2013; Franco et al., 2015). Strong flow/high shear stress seemed to attract endothelial cells making them polarize and migrate against blood flow (Tzima et al., 2002; Franco et al., 2015; Chang et al., 2017). Indeed, linear regression analysis identified a strong correlation between high shear stress values and cell polarization (Franco et al., 2015). But, how is blood flow and shear stress in the regressing segment? Quantification of blood flow and shear stress *in vivo* is challenging in particular in mouse models, and most information about shear stress values and patterns *in vivo*, in particular in capillaries, is derived from computational analysis and simulations. These models provide cues about these values in vascular networks with active capillary pruning as the mouse postnatal retina or the brain and the intersegmental vessels in zebrafish (Chen et al., 2012; Franco et al., 2015, 2016). In the postnatal mouse retina simulations predicted shear stress values up to 200 dyn/cm^2^ at arterioles, 100–150 dyn/cm^2^ at venules and 200 dyn/cm^2^ in normal capillaries to values near 0 dyn/cm^2^ in regressing capillaries which correlated with the percentage of cells aligned and polarized in each of these territories (Franco et al., 2015). Indeed, although dynamic quantification of shear stress cannot be analyzed, comparison of simulated distribution of blood flow and shear stress between P5 and P6 neonatal retinas suggest the presence of spatial and temporal gradients that could underlie pruning events (Bernabeu et al., 2014). Moreover, in the brain zebrafish vasculature capillaries with blood flow below a certain value were selected to regress (Chen et al., 2012). Table 1 shows shear stress values observed or simulated in the context of vessel regression. Of note, live-imaging in zebrafish brain vasculature demonstrated that regressing vessel segments exhibit low or reciprocating flow, which decreased irreversibly prior to the onset of regression (Chen et al., 2012). The shear stress threshold to enter the regression program seems variable and depends on relative levels on juxtaposed vessel segments indicating the need for spatial shear stress gradients (Chen et al., 2012). In relation to this, vascular segments with lower shear stress segments in a range between 2.5 and 10 dyn/cm^2^ often contained endothelial cells with very low axial or misaligned polarity vectors in the intersegmental vessels of zebrafish (Franco et al., 2015). However, in this state, endothelial cells were able to sense the onset of flow and responded dynamically polarizing against the direction of flow and migrating toward the highly perfused neighboring vessel. This paradoxical phenomenon, also observed in capillary splitting, underscores the relevance of shear stress ramping and gradients to trigger endothelial cell responses leading to microvascular remodeling. Finally, it has been observed that pruning of capillary segments often occurs at locations with H or O (loop) geometries, usually in flat vascular networks, but how bifurcations and intersections affect shear stress and thus endothelial cell migration and how capillary pruning may occur in 3D complex networks remains poorly characterized.

### Blood Flow and Capillary Splitting

Tiny holes representative of intraluminal pillars during intussusceptive angiogenesis were visible in vascular segments that were dilated and at triple or quadruple branching points in several tissues and organs (Patan et al., 1992), already indicating that blood flow and shear stress magnitude or gradients were relevant for this remodeling process. Indeed, a sustained increase in blood flow of between 50 and 60% in the chorioallantoic membrane of chicken was reported to increase shear stress and stimulate the creation of intraluminal pillars, leading to the division of the vascular sinus (Burri et al., 2004). Moreover, the increased vascular volume in the skeletal muscle induced by exercise or the administration of vasodilators as prazosin also led to capillary splitting (Zhou et al., 1998). In the zebrafish caudal vein plexus, blood flow is an essential requirement for vessel duplication as demonstrated by its occurrence only in perfused areas and by enhanced or reduced pillar formation observed with pharmacological strategies that increase (adrenergic agonist) or decrease (muscle contractility inhibitor) blood flow (Karthik et al., 2018). In models of experimental colitis, vasodilation of the intestine feeding arterioles has been reported (Mori et al., 2005) and impairing vasodilation prevents capillary splitting in the inflamed intestine (Esteban et al., 2020). Therefore, increased blood flow is considered a prerequisite for initiation of capillary splitting aimed to restore blood flow and shear stress in the duplicated segments (Egginton, 2011). Indeed shear stress will promote cellular events such as loss of apical-basal endothelial cell polarity, cytoskeleton and membrane rearrangements and filopodia protrusion to form the intraluminal pillars (Karthik et al., 2018) (Figure 2B). Although this review is focused on the role of shear stress in capillary remodeling, it is worth mentioning that increased blood flow may trigger splitting by elevating not only shear stress but also circumferential wall stress, which is similarly sensed by capillary endothelial cells (Lu and Kassab, 2011). How the increased blood flow induces the endothelial cell intraluminal rearrangements needed for the formation of pillars is far from being understood yet.

Since blood flow and shear stress cannot be quantified in the capillaries of the mucosa vascular plexus in the intestine, computational modeling has helped understand their contribution to capillary splitting. Previous computational studies of shear stress maps indicated that formation of new intravascular pillars was limited to regions of lower shear stress, less than 1 dyn/cm^2^, that were constrained between high shear stress in the pillar midportion and the lateral vessel wall (Filipovic et al., 2009) (Table 1). In parallel, flow modeling showed that these pillars were favored in areas with high flow (Szczerba et al., 2009). Consequently, the accepted paradigm is that development of the pillars is caused by increased flow but it occurs in low shear/turbulent flow areas (Djonov et al., 2002; Lee et al., 2010). This paradox (similar to capillary pruning) could be explained if the high flow was not continuous or whether it occurred after a period of decreased flow as observed in the rabbit ear (Styp-Rekowska et al., 2011). These observations would suggest a role for shear stress gradients and ramping in capillary splitting, so that, perhaps, sharp gradients of both shear stress and flow promote capillary duplication in areas which normally have very low shear stress. Indeed, this has been confirmed in an elegant study performed in zebrafish in which blood flow velocity and shear stress profiles have been simulated from live microscopy videos of the caudal vein plexus (Karthik et al., 2018). The authors observed that there was a steep drop in shear stress (about 2.5–5 dyn/cm^2^) in the region of pillar formation and initial fusion and then, as the pillar grows and splitting occurs, shear stress increased gradually (between 10 and 12.5 dyn/cm^2^).

### Beyond Blood Flow in Capillary Remodeling

At first, it was unclear how hemodynamic forces promoted endothelial cell migration in the mouse yolk sac (Udan et al., 2013), but it was proposed that endothelial cells could be responding to shear stress or substrate tension gradients (mechanotaxis) (Chiu and Chien, 2011) and/or to chemical signals released by cells sensing the force (chemotaxis). In microvascular remodeling, previous reports indicated that although blood flow is a critical driver, it needs to be coupled with other physical, chemical and cellular cues (Georgieva et al., 2019).

In the context of capillary pruning in the mouse postnatal retina, computational modeling studies stated that soluble signals such as VEGF (induced in low perfused regions) will complement blood flow actions in the vascular regression process (Watson et al., 2012). In addition to its role in optimizing network functionality, microvascular remodeling also aims at preventing the formation and persistence of preferential arteriovenous shunts which would negatively impact oxygen distribution (Pries and Secomb, 2014). In computational simulations during the remodeling of pillars into a differentiated network, it was predicted the need of a chemical inhibitor to avoid arteriovenous shunts (Szczerba et al., 2009). More recently, blood flow has been shown to act in coordination with endothelial cell collective migration for maintenance or avoidance of bifurcations which together with molecular signals and network geometry will contribute to capillary remodeling (Edgar et al., 2020).

In mouse experimental colitis, computational models pointed to the relevance of mechanical and chemical cues such as oxygen, metabolites and growth factors to fully explain capillary splitting together with blood flow and shear stress (Szczerba et al., 2009). It was proposed that tissue mechanical properties would regulate shear stress and lead to endothelial responses (recently reviewed in Gordon et al., 2020), but also to the secretion of chemical signals that will contribute to the splitting process and to the crosstalk with supporting cells (Szczerba et al., 2009; Egginton, 2011). These secreted chemical signals could promote or inhibit pillar growth fine-tuning the remodeling of the network. Most interestingly, they predicted the properties of such soluble factors which should have a diffusion coefficient between 10^–9^ and 10^–12^ m^2^/s and a molecular mass of 1 to 100 kD. The influence of mechanical tension on the process of capillary splitting has directly been demonstrated in the chorioallantoic membrane in which stretching stimulated capillary splitting (Belle et al., 2014).

Interstitial flow also leads to shear stress and other pressure forces on blood vessels that endothelial cells are able to sense and that may be contributing to some extent to the remodeling of the vascular tree. The role of interstitial flow has mostly been studied in the context of vascular morphogenesis and sprouting angiogenesis. However, although shear stress produced by interstitial flow is in the order of 10^–3^ dyn/cm^2^ (Rutkowski and Swartz, 2007; Shirure et al., 2017), orders of magnitude below those produced by blood flow, significant endothelial cell elongation and actin filament rearrangement has been reported for shear stress between 5.15 × 10^–2^ and 2.15 × 10^–1^ dyn/cm^2^ (Vickerman and Kamm, 2012), suggesting that interstitial flow may also contribute to capillary remodeling events. The effect of interstitial flow through the vessel wall (transmural) or around the vessel wall through the surrounding endothelial cells has been analyzed in different angiogenic contexts and in combination with VEGF actions (Song and Munn, 2011; Vickerman and Kamm, 2012). Interstitial flow was concluded to drive endothelial cell migration toward vessels that have higher microvascular pressure, a phenomenon reminding of capillary regressing events. Another relevant effect is the elimination of morphogen gradients since physiological values of interstitial fluid could dissipate morphogen gradients within hours in a magnitude-dependent manner (Shirure et al., 2017). Through these actions on endothelial cell migration and on the distribution of the biomolecular endothelial cell environment, interstitial flow may constitute an underestimated player in capillary remodeling.

### Common Features in Capillary Pruning and Splitting

Therefore, both modes of capillary remodeling rely on blood flow forces. In particular, on spatial-temporal gradients of shear stress established at particular network geometries as the intersections between capillaries and adjacent larger vessels (in pruning) or between adjacent capillaries (in splitting). These gradients coupled with capillary caliber changes drive endothelial cell rearrangements leading to cell migration toward the high flow/shear stress region either the adjacent highly perfused larger vessel (in pruning) or the lumen (in splitting). In addition, although both pruning and splitting events occur at sites of local lower shear stress, the observations point to high blood flow as the main driver, indicating that dynamics and ramping of shear stress changes are critical drivers. Finally, chemical signals as oxygen or VEGF and mechanical cues as tissue stiffness or interstitial flow can also contribute to both capillary pruning and splitting.

The co-existence of both remodeling processes can be envisioned in the entity called intussusceptive pruning in which pillar extension occurs in non-axial directions (Mentzer and Konerding, 2014); this has been observed in the extraembryonic vessels of chick embryos (Lee et al., 2011) and at the branch angles of bifurcating vessels in experimental mouse colitis (Ackermann et al., 2013). Moreover, reported images of the vascular plexus behind the sprouting front in the postnatal mouse retina show holes that could remind of intraluminal pillars (Turner et al., 2017), supporting the possible co-occurrence of both remodeling processes in this capillary plexus. Nevertheless, capillary splitting and pruning are still often considered independent and distinct processes. However, common hemodynamic regulatory cues together with the subsequent changes in the number and perfusion of microvascular segments lead us to propose that pruning and splitting could be the two sides of the same coin. That is, two different mechanisms of microvascular remodeling that are coordinated in time and space in response to changes in tissue oxygen demand and in blood flow distribution with the final aim to achieve an optimized and refined vascular network. It would not be unexpected that molecular mechanisms are also shared.

## Mechanisms in Blood Flow-Driven Capillary Remodeling

Shear stress sensed by endothelial cells induces different cellular responses that would finally lead to capillary remodeling *in vivo* (Davies, 1995; Wang et al., 2013). *In vitro* studies showed firstly that endothelial cells elongate and align in a shear stress-dependent manner with alignment requiring longer exposure times (Levesque and Nerem, 1985), and secondly that the higher the laminar flow and shear stress the larger the proportion of endothelial cells that polarize and migrate against flow direction (Table 2). These findings would suggest that endothelial cell elongation, alignment and polarization and migration against blood flow are sequential responses to higher and longer shear stress values, but the reality is more complex and depends on the endothelial cell type, the onset of blood flow (*in vitro* is usually abrupt) and the kinetics of the process under investigation (Dieterich et al., 2000) (Table 2). And what is it known about values of shear stress and endothelial cell responses *in vivo*? Without disregarding the potential effect of vessel caliber and geometry as well as matrix composition in endothelial cell elongation and orientation *in vivo* (Campinho et al., 2020; Gordon et al., 2020), previous reports (Tkachenko et al., 2013; Franco et al., 2015, 2016; Chang et al., 2017; Poduri et al., 2017) and our own observations indicate that endothelial cell elongation and polarization against the flow direction positively correlate with the shear stress magnitude estimated in different arterial beds and in capillaries during pruning (Table 1). These findings emphasize the influence of spatiotemporal regulation of blood flow/shear stress and the possible existence of threshold shear stress values for differential endothelial cell responses *in vivo* (Table 2).

**TABLE 2:** Shear stress-induced endothelial cell responses.

EC Type	SS (dyn/cm^2^)	Exposure Time	EC response	Comments	References
**Elongation**					
HUVEC	10	12 h	Elongation	Enhanced at 24 h	Steward et al. (2015)
HUVEC	20	12 h	Elongation		Ohta et al. (2015)
HAEC	10		Elongation		Mack et al. (2017)
	26				
BAEC	15.2	3 h	Elongation		Galbraith et al. (1998)
BAEC	30	24 h	Elongation		Levesque and Nerem (1985)
**Alignment**					
HUVEC	20	24 h	Alignment		Ohta et al. (2015)
HMVEC	9	21 h	Alignment	Non-oriented EC near the stagnation point and parallel to flow far from the center	Ostrowski et al. (2014)
	34 and 68			Azimuthal EC orientation at radial distances and parallel to flow far from SS peak	
	210			EC detachment near the flow orifice, and remaining EC with azimuthal orientation	
	Impinging			Model of Impinging flow	
MAEC	15	12 h	Alignment		Magid et al. (2003)
PAEC	<12		No alignment	Low effect on orientation	Dieterich et al. (2000)
	68		Alignment	Orientation within 10 min	
**Polarization (against flow unless indicated)**					
HUVEC	3	15 min	Polarization	≈50% subconfluent EC polarized (lamellipodia in flow direction)	Wojciak-Stothard and Ridley (2003)
HUVEC	20	4 h	Polarization	More than 60% of cells polarized	Franco et al. (2016)
HUVEC	Static and 4	24 h	Random orientation		Sonmez et al. (2020)
	7.2	3 h	Polarization	Different time of exposure	
	4.4, 18.6, and 40.2	24 h	Polarization	95% polarized (against flow at higher SS values, 18.6 and 40.2)	
HCAEC	14	24 h	Polarization	70–80%	Poduri et al. (2017)
**Migration**					
HUVEC	7.5	24 h	Migration	Smooth migration and long distances with flow vs pulsatile flow or static	Blackman et al. (2002)
HMVEC	9, 34, 68, and 210 Impinging flow	21 h	Migration	Faster migration at higher flow up to 68 dyn/cm^2^. At 210 dyn/cm^2^, pushed outward and then adapt, change direction, and migrate upstream after ∼16.7 h	Ostrowski et al. (2014)
HCAEC	35	72 h	Migration	Most migrating against the flow direction	Poduri et al. (2017)
**Other cellular responses**					
HUVEC	≈0.5 + 4 Reciprocating		Round shape Random and short actin filaments at periphery Slow migration High permeability	Model of reciprocating flow	Chiu and Chien (2011)
	>10 Laminar		Alignment	Compared with the reciprocating flow model	
			Long and parallel stress fibers at center		
			Fast migration		
			Low permeability		
MAEC	± 15 Reciprocating	12 h	No alignment	Model of reciprocating flow	Magid et al. (2003)
BAEC	0.5 ± 4 Reciprocating		Discontinuous VE-cadherin (similar to disturbed flow)	Model of reciprocating flow	Chien (2008)
BAEC	15.2	3 h	Thicker junctions		Galbraith et al. (1998)
			More stress fibers		
			More apical F-actin		
		6 h	MTOC and nuclei reorganization		
RFPEC	15	30 min	Filopodia protrusion		Zeng and Tarbell (2014)
PAEC	15	8 h	F-actin reorganization		Noria et al. (2004)

*Representative selected reports showing endothelial cell responses to distinct shear stress values and patterns *in vitro*. Values of shear stress correspond to laminar flow unless otherwise indicated. ECs, endothelial cells; HUVEC, human umbilical vein ECs; HCAEC, human coronary artery ECs; MAEC, mouse aorta ECs; BAEC, bovine aorta ECs; RFPEC, rat fat pad ECs; PAEC, pig pulmonary artery ECs.*

Molecular actors involved in blood flow-mediated endothelial cell responses, in particular the mechanosensor complex formed by PECAM-1/VE-cadherin/VEGFR2 at the junctions, have already been reviewed (Korn and Augustin, 2015; Campinho et al., 2020; Gordon et al., 2020). In this review, we will comment only some of the molecular pathways known to be regulated by shear stress and that seem to be relevant to endothelial cell responses in the context of blood flow-driven pruning and splitting (Tables 3, 4).

**TABLE 3:** Shear stress regulation of molecular effectors.

EC type	SS (dyn/cm^2^)	Exposure time	Molecular response	References
**Mechanosensors**				
HUVEC	20	10 min	Increase Piezo 1-dependent Ca^2^ peaks	Li et al. (2014)
MAEC	15		Piezo 1-dependent alignment	Ranade et al. (2014)
HPAEC	15	20–50 min	Polarized Piezo 1 to leading edge	Ranade et al. (2014)
HPAEC	15	10 min	YAP nuclear translocation and acto-myosin reorganization	Nakajima et al. (2017)
BAEC	5		40% Kir2.1 current increase	Jacobs et al. (1995)
**Notch, Wnt, BMP**				
HAEC	20	24 h	Maximum Notch1 mRNA	Mack et al. (2017)
HAEC	26		Plateau Notch1 nuclear translocation and polarization	Mack et al. (2017)
HUVEC	20	4 h	Increased alignment against flow direction (in absence of Wnt5a/Wnt11)	Franco et al. (2016)
HUVEC	12	45 min	Smad1 translocation	Baeyens et al. (2016)
HUVEC	12	24 h	BMP9, Klf2, Klf4 expression	Baeyens et al. (2016)
HCAEC	15	24 h	Increased endoglin expression	Chu and Peters (2008)
HUVEC	12	15 min	Association endoglin/Alk1 and enhanced BMP9 sensitivity	Baeyens et al. (2016)
HUVEC	10–20		Smad1/5 maximally activated	Baeyens et al. (2015)
**Signal transducers**				
HCAEC	5		Decreased Dach1 expression (gradient SS maintains its expression)	Chang et al. (2017)
HUVEC	1.5 or 15	3 h	Increase in APJ protein (also after an acute change to higher flow)	Busch et al. (2015)
BAEC	3.5–35	25 min	Increase in Erk5 activity	Yan et al. (1999)
BAEC	12	20 min–2 h	Increase in Erk5 activity	Yan et al. (1999)
HUVEC	14	2 h	Increase in Erk5 activity (continuous, pulsatile or to-an-fro flow)	Shalaby et al. (2017)
HUVEC	22		Increase in Ins1,4,5P3 (0.5 up to 6 min)	Nollert et al. (1990)
HUVEC	0.4, 1.4, and 22	30 min	Decrease in PI, PE, PA at 10-30 s and increase in DAG, free arachidonate and Ins1,4,5P3. IP3 peak at 10 min	Bhagyalakshmi et al. (1992)
BAEC	12	30 min	Increased Rac1 activity at 30 min	Tzima et al. (2002)
BAEC	15	30 min	Polarized Rac1 activity	Shao et al. (2017)
Zebrafish Brain	Decreased blood flow		Increased Rac1 activity	Chen et al. (2012)
HUVEC	23	1–20 min	pp130Cas/Crk association	Okuda et al. (1999)
PAEC	20	5 min	Polarized decrease in pp130Cas in edge opposite to flow	Zaidel-Bar et al. (2005)
BAEC	12	5 – 60 min	No recruitment of Nck to VEGFR2 (in contrast to VEGF 10 ng/ml)	Wang et al. (2007)
**ECM-related**				
μvascular rat EC	14	4 – 8 h	Decreased MT1-MMP expression (in contrast to cyclic strain)	Yun et al. (2002)
HUVEC	5.3 + S1P		Increase in MT1-MMP activity and EC membrane recruitment (in 3D collagen matrices)	Kang et al. (2011)
HUVEC	13	2 h	Enhanced TSP1 secretion to ECM	Gomes et al. (2005)
Prazosin in muscle	Increases blood flow		Increased ECM TSP1 *in vivo*	Bongrazio et al. (2006)
Yolk sac	Flow restauration	30 min–4 h	Recovers Nrp1 arterial expression	le Noble et al. (2004)
Muscle	Increased blood flow		Increase in Npr1	Williams et al. (2006b)
Mouse EC	20	24 h	Nrp1 association to PLXND1 and VEGFR2 mechanosensor	Mehta et al. (2020)
**Soluble factors**				
Umbilical Vessels	24 vs 4	1.5, 3, and 6 h	Biphasic down, up and down VEGF regulation	Gan et al. (2000)
HUVEC	10 (orbital shaker)	72 h	Increased VEGF165/VEGFR2/pVEGFR2	dela Paz et al. (2012)
HUVEC	20	38 h	eNOs (not under pulsatile flow)	Voyvodic et al. (2012)
HUVEC	8, 2–8 (periodic, 15 min) and 12.4 (reciprocating)		Increase in nitric oxide synthesis (in contrast to turbulent flow (1.2 to 11.7 dyn/cm^2^)	Noris et al. (1995)

*List of selected reports showing the regulation of molecular effectors in endothelial cells by distinct shear stress values and patterns *in vitro* and *in vivo*. ECs, endothelial cells; HUVEC, human umbilical vein ECs; HCAEC, human coronary artery ECs; PAEC, human pulmonary artery ECs; MAEC, mouse aorta ECs; PTEC, pulmonary trunk ECs; BAEC, bovine aorta ECs; S1P, sphingosine-1-phosphate.*

**TABLE 4:** Molecular actors in capillary remodeling *in vivo*.

Capillary pruning		
**Mechanosensors**		
Piezo 1 YAP/TAZ K^+^ channel Kir2.1	LOF, probable decreased vessel regression in mouse retina Nuclear location required for vessel regression in zebrafish LOF, reduced EC alignment and vessel regression in mouse retina	Ranade et al. (2014) Nagasawa-Masuda and Terai (2017) Boriushkin et al. (2019)

**Notch, Wnt, BMP**		

Notch Non-canon Wnt Alk1 Endoglin Smad1/5	LOF, EC elongation and decreased capillary regression in mouse retina LOF, increased sensitivity to SS-induced regression *in vivo* LOF, hyper-vascularization and AV malformations in mouse retina LOF, EC shape changes (no alignment), directed migration and AV shunts LOF, reduced vessel regression and loop formation in mouse retina	Lobov et al. (2011), Mack et al. (2017) Franco et al. (2016) Larrivee et al. (2012) Jin et al. (2017); Sugden et al. (2017) Benn et al. (2020)
IFT88	LOF, premature and random vessel regression in mouse retina	Vion et al. (2018)
**Signal transducers**		
CDS2 Rac1	LOF, increased vessel regression in zebrafish and postnatal mouse retina LOF, EC migration *in vivo* and defective vessel pruning in zebrafish brain	Zhao et al. (2019) Chen et al. (2012)
**Soluble factors**		
VEGF	Predicted contribution to capillary pruning in mouse retina	Watson et al. (2012)
**Capillary splitting**		
**ECM-related**		
MT1-MMP TSP1	LOF, decreased vessel splitting during mouse colitis *in vivo* LOF, decreased vessel splitting in mouse colitis	Esteban et al. (2020) Esteban et al. (2020)
**Soluble factors**		
VEGF Nitric oxide/eNOS	Promotes vessel splitting in skeletal muscle and CAM LOF, reduces capillary splitting in the skeletal muscle and correlates with less capillary splitting in mouse colitis	Baum et al. (2010); Gianni-Barrera et al. (2013) Esteban et al. (2020), Williams et al. (2006a)

**Other vascular remodeling events**		

**Signal transducers**		
Dach1 ApelinR Erk5 Nck p130Cas	GOF/LOF, EC polarization, alignment and migration against flow and LOF, impaired embryonic arterial patterning Required for EC polarization *in vitro* and *in vivo* in zebrafish LOF, Disorganized and rounded ECs *in vivo* LOF, impaired EC front-rear polarity and VEGF-directed migration *in vivo*; no impact in vessel regression in retina LOF, less focal adhesion turnover and EC directed migration *in vitro*	Chang et al. (2017) Kwon et al. (2016) Nithianandarajah-Jones et al. (2014), Spiering et al. (2009) Chaki and Rivera (2013), Dubrac et al. (2016) Spiering et al. (2009), Zaidel-Bar et al. (2005)
**ECM-related**		
Nrp1	Enables EC filopodia via cdc42 in zebrafish and mouse retina and regulates EC shape, cell contacts, and actin in collective migration in zebrafish	Fantin et al. (2015), Hamm et al. (2016)

*Representative molecular players with reported or suggested actions in capillary regression, splitting or other vascular remodeling events *in vivo*. EC, endothelial cells; GOF, gain-of-function; LOF, loss-of-function.*

The new concepts in capillary remodeling predict that the molecular pathways related to sensing blood flow/shear stress by endothelial cells and its transduction into extracellular and intracellular gradients will be of special relevance to modulate the process. The non-canonical Wnt signaling pathway (Wnt11 and Wnt5a) regulates vessel regression in the mouse retina by affecting the sensitivity of the vasculature to shear stress (Franco et al., 2016). The role of BMP signals can be more complex since BMP9 cooperates with the primary cilium to prevent vessel regression under low shear stress (Vion et al., 2018) but the absence of endothelial BMP-SMAD1/5 signals results in reduced vessel regression with aberrant vascular loops and arteriovenous malformations in areas with high blood flow in the mouse retina (Benn et al., 2020). Endoglin, a TGFβ/BMP co-receptor, is required to couple flow-mediated mechanical cues with endothelial cell migration and shape modulating final vessel remodeling (Lobov et al., 2011; Boriushkin et al., 2019). The role of novel mechanosensors (in addition to the well-known PECAM-1/VE-cadherin/VEGFR2 complex) in driving polarized endothelial cell responses is also an active field of research (see review by Campinho et al., 2020). Among these mechanosensors, Piezo 1 increases intracellular calcium in response to shear stress and regulates endothelial cell migration via nitric oxide production and its absence leads to defects in embryonic vascular remodeling suggestive of defective pruning (Li et al., 2014; Ranade et al., 2014). Downstream of Piezo 1, the transcriptional factor Yap/Taz, responsive to both shear stress and endothelial cell stretching (Nakajima et al., 2017; Neto et al., 2018), has been proved to be active during capillary remodeling by inducing actin polymerization; this resulted in a decrease in vascular regression when Yap/Taz pathway is silenced (Nagasawa-Masuda and Terai, 2017). Endothelial K^+^ channel Kir2.1 has recently been identified as a shear stress sensor whose absence led to decreased endothelial alignment in retinal endothelium and reduced capillary pruning near the angiogenic front of postnatal retinas (Boriushkin et al., 2019). The quick response of these mechanosensors to shear stress (Table 3) underscores their role as initial upstream regulators of early endothelial cell responses as alignment and polarization required for capillary pruning (Table 4). Of interest is the transcription factor Dach 1 that is regulated by laminar flow specifically, being expressed in arteries subjected to low flow in which it stimulates endothelial cell migration against blood flow (Chang et al., 2017) suggesting a similar not-yet investigated role during capillary pruning. The actions of Notch pathway in capillary pruning seem more complex and pleiotropic; inhibition of Dll4/Notch was shown to prevent retinal capillary regression in the mouse retina by regulating vasoconstriction and blood flow (Lobov et al., 2011) and deletion or loss of its inhibitor Nrapr resulted in enhanced vessel regression, likely by the additional modulation of Wnt signaling (Phng et al., 2009). Moreover, since lower Notch activity correlated with more mobile VE-cadherin at endothelial junctions (Bentley et al., 2014a), differential Notch activity may coordinate endothelial cell arrangements during polarized migration in capillary regression (Franco et al., 2015). Accordingly, endothelial Notch1 was demonstrated to be responsive to shear stress and necessary for the maintenance of junctional integrity induced by laminar shear stress (Mack et al., 2017). However, shear stress sensing needs to be transduced into polarized intracellular signals to promote alignment, polarization (with the Golgi in front of the nucleus toward the migrating edge) and directed migration. In this line, the small GTPase Rac1, regulator of actin polymerization and cell migration, is required for vessel regression in the zebrafish brain (Chen et al., 2012) and polarized Rac1 subcellular gradients are induced by laminar (15 dyn/cm^2^) but not disturbed flow in endothelial cells *in vitro* (Shao et al., 2017). Similarly, phosphoinositide gradients are required for a balanced capillary pruning induced by VEGF since the absence of CDP-diacylglycerol synthase-2 (CDS2), a metabolic enzyme that controls phosphoinositide recycling, in mouse and zebrafish showed increased endothelial cell migration and vessel regression (Zhao et al., 2019). VEGF was considered an essential player in vascular regression occurring after deprivation of VEGF or VEGF-induced signals which led to endothelial cell apoptosis in different tissues and contexts (Meeson et al., 1999; Tang et al., 2004; Baffert et al., 2006). Previous *in vivo* and *in silico* analysis in the postnatal mouse retina pointed to VEGF as a complementary actor together with blood flow in capillary pruning in this context (Watson et al., 2012). However, how VEGF precisely participates in endothelial cell migration against blood flow during capillary pruning remains undefined.

Much less is known about the mechanisms underlying capillary splitting or duplication since there is no optimal *in vitro* model for its study and the endothelial cell processes involved are less understood. In a pioneer attempt to understand the molecular pathways involved in capillary splitting, Egginton’s group aimed at identifying genes differentially expressed in the context of capillary splitting (vasodilator-treated) versus sprouting (agonist-excised) in the skeletal muscle and they found that endothelial nitric oxide synthase (eNOS) and neuropilin-1 were upregulated (Egginton, 2011). Nitric oxide is a downstream target of the laminar flow-induced transcription factor Klf2 (Nayak et al., 2011) but also by shear stress-stimulated glycocalyx on endothelial apical side (Bartosch et al., 2017). Indeed, nitric oxide produced by the endothelial cells is essential not only to induce the endothelial cell rearrangements required for intraluminal pillar formation and splitting, in particular luminal filopodia protruding mostly from the inter-junctional regions, but for capillary splitting to occur as demonstrated in the skeletal muscle of vasodilator-treated eNOS-deficient mice (Baum et al., 2004; Williams et al., 2006a). A recent report from our group has added a layer to nitric oxide regulation showing that TSP1 cleavage by the protease MT1-MMP promotes endothelial cell production of nitric oxide favoring vasodilation and capillary splitting during experimental colitis (Esteban et al., 2020); whether in this model nitric oxide mainly acts by inducing vasodilation of the feeding arterioles or has additional actions in the endothelial cell rearrangements to form the pillars during capillary splitting still needs to be established. Of note, nitric oxide is a soluble factor with properties close to those predicted for the soluble factor required for refining capillary splitting according to *in silico* models (Filipovic et al., 2009). The role of VEGF in the process is supported by its expression in the chorioallantoic membrane microvasculature and the delay in capillary splitting when VEGF was inhibited (Baum et al., 2010) and by the induction at high doses of dysfunctional capillaries by splitting (Gianni-Barrera et al., 2013); VEGF actions in this context can be modulated by PDGFB and EphrinB2/EphB4 pathways (Gianni-Barrera et al., 2018; Groppa et al., 2018). Novel actors include the inhibitor of proteases RECK which regulates non-sprouting (likely splitting) vascular remodeling during embryonic development (Chandana et al., 2010) and Notch (Dimova et al., 2013) and endoglin, whose inhibition led to enhanced capillary splitting by distinct mechanisms (Hlushchuk et al., 2017).

Of course, the cellular and molecular understanding of capillary remodeling and its regulation is far from being complete. Other possible cues not much investigated yet, but with possible roles in regulation of endothelial cell morphology, include endothelial cell junction components that may be a critical factor to explain rearrangements during both regression and splitting. In particular, VE-cadherin forms part of the mechanosensory complex together with PECAM-1 and VEGFR2 (Tzima et al., 2005) and, it has been reported that short-term reciprocating flow reduces VE-cadherin localization at the junctions while sustained exposure to pulsatile flow reinforces it (Miao et al., 2005). Since reciprocating flow is observed at the regressing segment (Chen et al., 2012), subsequent reduction in junctional VE-cadherin may favor destabilization of endothelial junctions and allow endothelial cell migration to the adjacent high flow vessels. Cues from the basement membrane on which directed endothelial cell migration takes place toward the adjacent vessel can also be important as suggested by recent studies with gradients of a collagen IV peptide *in vitro* (Du and Gao, 2019). Actin cytoskeleton may be an additional actor since short exposure to laminar flow induces apical F-actin reorganization and filopodia (Galbraith et al., 1998; Zeng and Tarbell, 2014) (Table 2) and actin filaments are visualized in the pillars during capillary splitting although their ability to generate the required force for intraluminal protrusions under blood flow is still unclear (Paku et al., 2011; Mentzer and Konerding, 2014). Finally, other players in endothelial directed migration as the Apelin GPCR (Kwon et al., 2016), the actin cytoskeleton regulators cdc42 (Lavina et al., 2018) and Nck (Chaki and Rivera, 2013), the atypical MAP kinase Erk5 (Spiering et al., 2009), the TGFβ/BMP receptor Alk1 (Rochon et al., 2016) or the adapter p130Cas (Evans et al., 2017) could also be good candidates to participate in capillary remodeling *in vivo* (Tables 3, 4).

## New Methodological Approaches and Tools to Understand Capillary Remodeling *in vivo* and *in vitro*

It is clear that capillary remodeling events depend largely on local spatiotemporal gradients of blood flow, but also cellular gradients (molecular signals) and luminal and tissue gradients (soluble factors), which forces us to change the paradigm of how to analyze cellular and molecular actors involved in these processes. Remodeling cannot be analyzed in bulk approaches but at single cell level as also recently reviewed (Campinho et al., 2020). This need demands advanced imaging techniques (with reporters and probes to track possible actors and signals) *in vitro*, *ex vivo*, and *in vivo* but also screening for new players by single-cell and spatial techniques for protein changes and modifications since some of these events occur in a time-scale that would not allow major gene changes (Lundberg and Borner, 2019).

### Single-Vessel and Single-Cell Image Analysis

Although *in vitro* models have provided relevant cues about cellular and molecular mechanisms involved in endothelial cell responses to changes in blood flow gradients, *in vivo* models allow a closer look at the endothelial cell behavior in the real physiological context. *In vivo* models used in the context of microvascular remodeling have typically focused on either immature microvascular networks during development or in pathological scenarios. Most advances have been made by the use of animal models such as the postnatal mouse retina (Connolly et al., 1988) and the intersegmental vessels and the brain vasculature in the transparent zebrafish (Chen et al., 2012; Franco et al., 2015) for segment pruning and the choriollantoic membrane of the chicken (Feinberg et al., 1986), the caudal venous plexus of the zebrafish (Karthik et al., 2018), the skeletal muscle (Hudlicka et al., 2006) and the experimental mouse colitis (Esteban et al., 2020) for capillary duplication.

Once endothelial cell migration triggered by changes in the blood flow sensing was established as the main actor in the vascular remodeling, markers implicated in the migration of the cells became an ideal target to monitor the behavior of these cells and also identify the potential vessels involved in this process. Thus, transgenic reporter animals became an essential tool in the investigation of vascular remodeling. Transgenic lines carrying endothelial cell fluorescence reporters have allowed live-imaging and visualization of the dynamics of the process in zebrafish (Scheer and Campos-Ortega, 1999; Lawson and Weinstein, 2002; Chen et al., 2012). More recently, Franco’s group has generated the GNrep transgenic mouse line with a double fluorescence reporter for the Golgi apparatus and the endothelial cell nucleus which allows a direct analysis of endothelial cell polarization in vascular territories undergoing remodeling (Barbacena et al., 2019). Notably, the use of the Raichu Rac1 FRET sensor established the role for the activity of this small GTPase in regulating directed endothelial cell migration during capillary pruning in the brain vasculature of zebrafish (Chen et al., 2012). LifeAct reporter has recently been used for visualization of endothelial cell filopodia and dynamics in the postnatal mouse retina *ex vivo* (Riedl et al., 2008; Prahst et al., 2020) and it could be a useful tool to analyze cytoskeletal rearrangements of endothelial cells during capillary remodeling. A better knowledge of the molecular actors involved in each step of segment pruning or splitting could help designing new fluorescent reporter animal lines.

**FIGURE 3:**
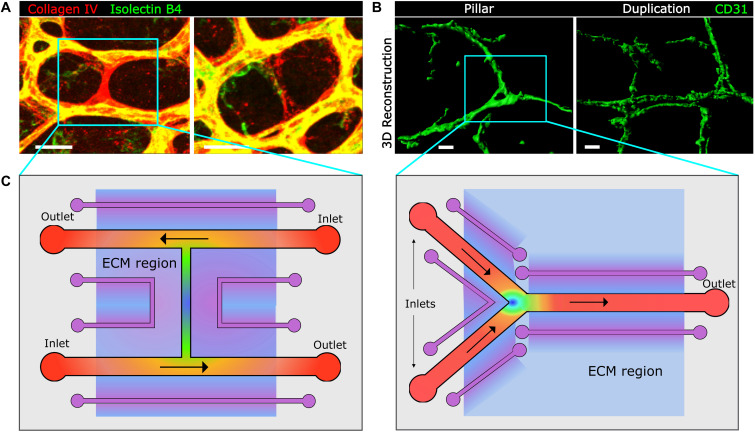
Image-based approaches to capillary remodeling *in vivo* and proposed device-based simulation *in vitro*. **(A, B)** Examples of whole-mount staining and confocal microscopy for the visualization and analysis of capillary pruning in the mouse postnatal retina **(A)** and of capillary splitting in the inflamed mouse intestine by means of 3D reconstruction with Imaris software. Scale bar, 20 μm. **(C)** Top view of proposed microfluidic chips for the study of capillary regression in an H-type geometry (left panel) and of capillary splitting in Y-type geometry (right panel), mimicking the geometries found in microvascular networks (blue boxes in panels **A** and **B**). Inlets and outlets for flow are indicated and are exchangeable depending on the preferred direction of flow in the different segments. Vessel channels are covered with a basement membrane and endothelial cells. In purple auxiliary channels for creating biochemical gradients along the extracellular matrix (ECM)-like hydrogel region (light blue). This hydrogel could also incorporate cells for co-culture analysis. Heat map color represent theoretical shear stress values (as in Figure 2) for established flow gradient profiles and shading represent biochemical gradients along the ECM. The rest of the microfluidic chip would be made of PDMS.

In addition to time-lapse microscopy in amenable models as zebrafish, confocal microscopy and 3D image analysis have largely advanced our understanding of capillary remodeling events (Campinho et al., 2020). Confocal microscopy in the quasi-2D flat mount postnatal retina has allowed precise quantification and characterization of “empty sleeves” (collagen IV positive, endothelial cell negative), the hallmark of capillary pruning events (Figure 3A). The challenge of exploring capillary pruning in more complex 3D vascular plexus remains and novel confocal microscopy-based 3D image processing tools and algorithms may help comprehend this process (Gkontra et al., 2018, 2019). Likewise, confocal microscopy of vessel-stained tissues such as whole-mount inflamed intestine combined with 3D-image rendering has turned into an approach that provides sufficient resolution to identify and quantify capillary holes/pillars, loops, and duplications (Figure 3B) (Karthik et al., 2018; Esteban et al., 2020), all hallmarks of capillary splitting, circumventing the limitations of previously used corrosion cast techniques and electron microscopy (Nowak-Sliwinska et al., 2018). Light-sheet fluorescent microscopy (LSFM) has recently been applied to the quantitative 3D and 4D analysis of endothelial cell motility and filopodia dynamics in blood flow-free *ex vivo* postnatal mouse retina (Prahst et al., 2020). Advanced microscopy techniques will undoubtedly improve our knowledge about capillary remodeling in more complex vascular beds what combined with the use of fluorescent reporter animal lines may provide unprecedented visualization and information about these events.

Novel mathematical and computational tools can also be of great value to understand the changes and actions of blood flow and shear stress, specially their gradients and dynamic changes. Although continuous blood flow models can provide some information about blood flow fluctuations and drop pressure changes in the microvascular network by means of permeability tensors (Gkontra et al., 2019), capillary remodeling research will most benefit from discontinuous or segment-based models and simulations. This has been the base for the efforts made to simulate the complexity of the hemodynamic forces even in simple networks as the postnatal retina vasculature (Bernabeu et al., 2014) and the development of new tools such as PolNet (an open-source software; Bernabeu et al., 2018) which aimed at facilitating the combined analysis of blood flow and shear stress with cell polarization in order to understand their influence in capillary remodeling by comparison of values between neighbor segments and at different developmental stages. This tool is available and useful for the study of blood flow in low complexity network as retinas. It is worth mentioning that computational modelization of particles has just uncovered the essential role of circulating red blood cells in generating regional shear stress differences and thus in regulating vascular remodeling by modulation of blood viscosity (Zhou et al., 2020). Of note, new advances in mathematical approaches in the multidisciplinary field of Adaptive Systems could also help decipher the dynamic changes of endothelial cells responding to a variety of environmental signals in order to adopt the best network architecture possible (Bentley et al., 2014b).

### *In vitro* Systems for Analysis of Microvascular Remodeling Under Flow: Microfluidics as a Promising Technology

*In vitro* flow chambers could be considered the preludes of microfluidic-based devices as they mimic hemodynamic shear stress in macroscale flow chambers with 2D endothelial cell monolayers. The two main models of *in vitro* flow chambers are the parallel plate flow chamber and the cone and plate viscometer with its corresponding modifications; in both cases, cells are cultured on a two-dimensional stiff surface and fluid is flown over the culture. While the parallel plate system has several advantages as its simplicity or its compatibility with imaging modalities, the effects of shear stress cannot be discriminated from those due to hydrostatic pressure. On the other hand, the cone and plate viscometer does allow to separate both effects but it has other limitations as its difficult combination with imaging and the need of continuously supply the culture with fresh medium (Papadaki and McLntire, 1999). The most popular commercial versions of flow chambers are the family products Bioflux (multi-well format with small fluid reservoir) and Ibidi (varied plate formats with larger fluid reservoir for longer experiments) which are widely used among researchers in the field. They are relatively easy to implement and offer advantages as its high reproducibility, good cell viability and technical support on-hand. But they come with limitations since they are not very versatile (fixed geometries and fluid properties) and they are expensive (disposable and non-reusable plates and consumables) (Roest et al., 2011). These type of 2D systems are mostly interesting to examine the role of shear stress exerted by different types of flow including laminar, pulsatile and reciprocating flow (Usami et al., 1993; Chien, 2007) but, in addition to the limitations already discussed, they do not incorporate the remodeling of the extracellular matrix (ECM) nor its mechanical properties, failing to fully recapitulate the complex 3D environment, geometries or the shear stress gradients found *in vivo* (Coluccio et al., 2019; Gordon et al., 2020).

Microfluidic chips are characterized by having channel sizes of tens to thousands of micrometers and dealing with volumes in the microliter to picoliter range; in fact, they are considered such when one or more channels have at least one dimension smaller than 1 mm. Switching from the macroscale to the microscale offers new advantages for researchers including a big reduction in the amount of reagents but more importantly a huge versatility for exploring new geometries and flow conditions (van Duinen et al., 2015). The main advantages of this technology are that it provides the possibility of isolating and controlling fluid properties, physical, chemical and biological stimuli while being compatible with several imaging modalities including time-lapse microscopy (Hsu et al., 2013). They can also be used in a high throughput manner so several conditions can be tested simultaneously (Hsu et al., 2013). Among the applications of interest in the miocrovascular remodeling field is the analysis of vascular cell responses to shear stress (Wong et al., 2012). Even though microfluidic technology is usually applied for quasi-2D models with laminar flow, in the last years new techniques implemented fully 3D models that offer a more physiological environment.

Microfluidic devices are typically built in PDMS (polydimethylsiloxane) because being it transparent, permeable to oxygen and compatible with soft-lithography and rapid prototyping techniques, it is a good option to build biocompatible devices for research with custom-made geometries. Nevertheless, further demands for deeper understanding of vascular phenomena made PDMS-hydrogel hybrid devices emerge as a better option for the study of angiogenesis and vascular remodeling (Bogorad et al., 2015; Gordon et al., 2020). This type of devices incorporates a hydrogel region surrounding the endothelial cell-covered microchannels which can be remodeled by cells and whose mechanical properties resemble much better the endothelial microenvironment. In addition, with the hydrogel regions, different types of biochemical gradients can be incorporated throughout the device. The possibility to control and model computationally both the biochemical and physical gradients (Nguyen et al., 2013) and the flow and shear stress distribution, timing and ramping along the device (Chiu and Chien, 2011) makes it possible to establish correlations between the different types of stimuli and the endothelial cell response. Given that intraluminal endothelial migration occurs in contact with the preserved basement membrane during capillary remodeling, it is worth mentioning that microfluidic systems including a basement membrane have been implemented for the study of endothelial cell permeability or cancer metastasis (Han et al., 2015; Kim et al., 2019; Coughlin and Kamm, 2020).

One drawback of lithographic techniques commonly used to produce the PDMS structure is the limitation to rectangular cross-sections rather than the natural cylindrical shape (Akbari et al., 2017), that could influence the outcome of the experiments due to pulling and traction forces at the corners of polygonal shapes (Rauff et al., 2019). Cylindrical cross-sections have been achieved with 3D microvessel templates by cross-linking the hydrogel around a needle or rod that is then removed. However, this technique is limited to linear geometries and larger diameters (60–200 μm). The report of a grid geometry for microvessel networks that resemble the highly branched native plexus, where blood passes from capillaries to arterioles and venules, could be of interest as a retina model since in both cases blood flow diverges and decelerates as it branches, and consequently it creates different shear stress patterns (Zheng et al., 2015). Going even further, fully hydrogel embedded systems that can incorporate other cell types such as pericytes in co-culture, are the most accurate at resembling the three-dimensional physiological microenvironment.

PDMS-hydrogel hybrid devices can incorporate branching and bifurcating regions of special interest for capillary splitting and other remodeling events due to the different flow patterns occurring at these sites. This type of devices have recently been used to produce impinging bifurcating fluid flow, laminar shear stress and transvascular fluid flow (Akbari et al., 2018) and also controllable disturbed flow patterns for the study of its effects on actin stress fiber and endothelial cell orientation (Tovar-Lopez et al., 2019). But how exactly does the direction, slope and average magnitude of shear stress gradients affect endothelial cell biological and molecular responses needs still to be understood. Due to the geometrical versatility and high reproducibility and control that microfluidic devices offer, this technology springs as a promising tool for a better understanding of these events. For example, a recent microfluidic device was able to produce areas with three different constant shear stress values and six different shear stress gradients and showed that human endothelial cell upstream orientation depends on gradient direction (Sonmez et al., 2020). Following a top-down approach (Hesh et al., 2019), we propose two prototypes of hybrid devices that on one hand are inspired on real microvascular networks in which capillary pruning and splitting occurs (Figure 3A,B), mimicking their preferential H and Y geometries; and on the other, they incorporate predicted shear stress values and spatiotemporal gradients, as discussed along this review, whose tuning and dynamics could only be recapitulated in these systems (Figure 3C). These prototypes could even represent better the *in vivo* microvascular remodeling context by including a physiologically relevant ECM, a basement membrane and supporting cells in co-culture.

Microfluidic devices offer several advantages. The influence of the environment on the microvasculature, in particular chemical factors, can be tested not only controlling its concentration but its gradients for example of WNTs, Notch-related molecules, VEGF, all with important roles in vessel pruning (Wong et al., 2012; Korn and Augustin, 2015). The possibility of time-lapse imaging allows to study the dynamics of microvascular remodeling events (Wong et al., 2012). Some devices can incorporate supporting cells such as pericytes (Rauff et al., 2019), contributors to microvascular remodeling. *In vitro* microfluidic models designed using solely human components may serve as a translational bridge between experimental animal models and human applications given the much larger shear stress values found in mouse compared to human vessels (Chiu and Chien, 2011; Zheng et al., 2015). Microfluidic models can also offer a platform to test and refine computational models. Mathematical and computational models require tight boundary conditions (like flow parameters at the inlet and outlet or pre-defined values at certain locations) that are impossible to control or measure *in vivo*. Since flow parameters can be tuned by the user in microfluidic chips and they are compatible with several imaging modalities and measuring techniques, microfluidic platforms could be a great complement to feed these theoretical models.

A bit aside from the classical microfluidic chips that have been described so far, but that may be relevant in the field, are organ mimetic microdevices and organ-on-a-chip devices. These technologies combine microfluidic technology with other culture systems as bioreactors and microchambers and computer control systems (Huh et al., 2011).

Even though the advantages of microfluidics versus macroscale flow chambers are vast, the fact that it has recently started to be exploited for biological research poses some difficulties related to their implementation by researchers. Protocols for biochemical assays and cellular cultures are established for macroscale conditions where the surface to volume ratio is small and the culture media to cell ratio is large. These conditions have to be carefully adjusted to the microscale to avoid artifacts and cell death and comparison with the results obtained in macroscale systems and classical biochemical assays have to be made with care. For PDMS devices, attention has to be paid with the surface coating for proper cell attachment (which is different to that in glass or polystyrene). Also, CO_2_ concentration and pH needs to be checked since, even though it is permeable to gases, too thick PDMS layers can hinder proper ventilation of the culture. The perfusion system (that can be gravity-driven, external syringes or on-chip peristaltic pumps, all of them having been proved to allow more than 1 week perfusion) has to be carefully configured to ensure both adequate nutrient flow at the flow rate required for the shear stress pattern to be studied and bubble-free fluid flow (Kim et al., 2007). Fluid control can become specially challenging in self-organized microvessels due to their random nature. Lastly, it has to be mentioned that often microfluidic control systems are rather complex to use and researchers may not be familiar with its handling (Halldorsson et al., 2015).

All in all, we believe that the microfluidic technology needs still to be exploited to reach its full potential in the study of vascular remodeling events because of the many advantages it offers in comparison to other *in vitro* systems and that standardization of this type of experiments will overcome many of the difficulties it may pose nowadays.

## Conclusions and Perspectives

Tissues continuously adjust their oxygen and nutrient demands and for that they require the dynamic adaption of the microvasculature in order to optimize blood flow distribution. This is achieved by means of capillary remodeling, eliminating poorly functional or redundant segments by pruning, or duplicating segments to expand the microvasculature by splitting. Defective or excessive capillary remodeling will deprive tissue regions of appropriate perfusion leading to tissue damage or limiting its repair. This process has therefore attracted a lot of recent interest not only to understand vascular homeostasis but also to propose new strategies devoted to prevent disease and promote tissue repair (Georgieva et al., 2019).

Active research on the basic mechanisms of capillary remodeling beyond blood flow-driven endothelial cell migration can help identifying novel actors and modulators with potential use in preventing or ameliorating diseases related to excessive or defective capillary splitting or pruning as for example inflammatory bowel diseases, lung dysplasia, myocardial infarction or Alzheimer’s disease among others. As an example, the small GTPase Rac1 has been involved in capillary pruning in the brain zebrafish microvasculature and it is tempting to speculate that Rac1 inhibitors may help prevent excessive brain capillary pruning associated for example with aging or Alzheimer’s disease (Garkavtsev et al., 2011; Chen et al., 2012). On the other hand, excessive capillary splitting has been shown to contribute to the progression of inflammatory bowel disease and targeting recently identified actors as the molecular axis MT1-MMP/TSP1/eNOS by delivery of inhibitory antibodies or competitive peptides was shown to ameliorate the progression of the disease in mice (Esteban et al., 2020). Since failed microvascular remodeling can also lead to the presence of undesired arteriovenous shunts, recent identification of endoglin as a key regulator of endothelial cell responses for preventing AVM, can shed light about mechanistic links between endothelial cell responses to blood flow and disease. Furthermore, provocatively considering capillary pruning and splitting as two coordinated processes which continuously translate blood flow/shear stress fluctuations into microvascular adaptations can also help advance our understanding of these vascular events.

Finally, research efforts should be encouraged for the development and implementation of approaches and tools which more closely recapitulate the dynamics, geometries and flow conditions and the biochemical microenvironment of *in vivo* capillary remodeling in order to guarantee a wider comprehension of this process in near future.

## Author Contributions

RS and SE obtained microscopy images. RS and MG-Á prepared the figures. RS, MG-Á, and AGA wrote the text and all authors revised it. All authors contributed to the article and approved the submitted version.

## Conflict of Interest

The authors declare that the research was conducted in the absence of any commercial or financial relationships that could be construed as a potential conflict of interest.
